# Modelling Conditions and Health Care Processes in Electronic Health Records: An Application to Severe Mental Illness with the Clinical Practice Research Datalink

**DOI:** 10.1371/journal.pone.0146715

**Published:** 2016-02-26

**Authors:** Ivan Olier, David A. Springate, Darren M. Ashcroft, Tim Doran, David Reeves, Claire Planner, Siobhan Reilly, Evangelos Kontopantelis

**Affiliations:** 1 Institute of Biotechnology, University of Manchester, Manchester, United Kingdom; 2 Centre for Primary Care, NIHR School of Primary Care Research, Institute of Population Health, University of Manchester, Manchester, United Kingdom; 3 Centre for Biostatistics, NIHR School of Primary Care Research, Institute of Population Health, University of Manchester, Manchester, United Kingdom; 4 Centre for Pharmacoepidemiology and Drug Safety, Manchester Pharmacy School, University of Manchester, Manchester, United Kingdom; 5 Department of Health Sciences, University of York, York, United Kingdom; 6 Division of Health Research, University of Lancaster, Lancaster, United Kingdom; 7 Centre for Health Informatics, Institute of Population Health, University of Manchester, Manchester, United Kingdom; Indiana University, UNITED STATES

## Abstract

**Background:**

The use of Electronic Health Records databases for medical research has become mainstream. In the UK, increasing use of Primary Care Databases is largely driven by almost complete computerisation and uniform standards within the National Health Service. Electronic Health Records research often begins with the development of a list of clinical codes with which to identify cases with a specific condition. We present a methodology and accompanying Stata and R commands (*pcdsearch/Rpcdsearch*) to help researchers in this task. We present severe mental illness as an example.

**Methods:**

We used the Clinical Practice Research Datalink, a UK Primary Care Database in which clinical information is largely organised using Read codes, a hierarchical clinical coding system. Pcdsearch is used to identify potentially relevant clinical codes and/or product codes from word-stubs and code-stubs suggested by clinicians. The returned code-lists are reviewed and codes relevant to the condition of interest are selected. The final code-list is then used to identify patients.

**Results:**

We identified 270 Read codes linked to SMI and used them to identify cases in the database. We observed that our approach identified cases that would have been missed with a simpler approach using SMI registers defined within the UK Quality and Outcomes Framework.

**Conclusion:**

We described a framework for researchers of Electronic Health Records databases, for identifying patients with a particular condition or matching certain clinical criteria. The method is invariant to coding system or database and can be used with SNOMED CT, ICD or other medical classification code-lists.

## Introduction

The use of Electronic Health Record (EHR) databases is becoming more commonplace in medical and health services research. Development and use of EHR repositories is now established in the US, Canada and Scandinavia.[1–3] The UK has been leading on the development of such repositories, particularly Primary Care Databases (PCDs), with several large and many smaller databases currently in use.[4] This can be attributed to two UK-specific conditions that have favoured such a development. First, the umbrella of a single National Health Service (NHS), using broadly uniform health care procedures across providers. Second, the near-universal adoption by general practices of clinical computer systems with defined interoperability specifications, facilitated by government subsidies and the prospect of participating in a lucrative nationwide incentivisation scheme that required computerisation.[5] These databases are a potentially valuable resource for researchers if carefully analysed,[6, 7] but they have not enjoyed universal acceptance in the research community, due in part to the observational nature of the data.

All UK primary care clinical systems use coding systems to record clinical information. Hierarchical ‘Read’ codes are commonly used to record symptoms, diagnoses and referrals, although clinical systems also allow narrative or free-text entries for consultations Well-coded data entries record information that can be stored in a relational database and is therefore easily retrievable for the benefit of the patient, either directly during a consultation (allowing the clinical computer system to function as a decision support tool), or indirectly by secondary analysis of the data in the context of PCD research. Unfortunately, code use has not been consistent over time and variation in coding behaviour between practices is evident. In 2004 the Quality and Outcomes Framework (QOF) financial incentivisation scheme was introduced, rewarding practices for achieving quality targets across a range of chronic conditions. Centrally determined code lists (“business rules”) were created to identify patients with relevant conditions and to record achievement of quality targets, and as a result coding for conditions included in the QOF became more uniform and complete.

This was particularly noticeable for conditions that were previously poorly recorded. For example, in 2005/6, recorded prevalence rates for chronic kidney disease (CKD) in patients with severe mental illness (SMI) and in control groups were 1.0% and 0.6% respectively, but increased to 5.5% and 3.2% in the following year when CKD was incorporated into the QOF, and increased to 8.2% and 4.2% by 2011/12.[8] Although underlying prevalence of CKD is estimated to be increasing,[9] the step-change in prevalence in 2006/7 is likely to be attributable to changes in recording and coding practice. Further increases in prevalence rates over time, especially for SMI patients, are likely to be due to better case finding, directly or indirectly driven by the QOF.[10]

It therefore seems likely that the quality of recording for QOF conditions (of which there were 17 in 2011/12) will be more reliable than for conditions not incentivised under the scheme. When investigating a QOF condition, it also seems reasonable to use the QOF business rules as a starting point (available from www.clinicalcodes.org). However, researchers might be interested in a broader definition of the condition, or may aim to measure activity in the pre-QOF period. In this case the QOF business rules alone might be inadequate, and a process is required that ensures the creation of a code-list that is as reliable and as inclusive as possible, suitable to answer the specific research question. Clinical code lists lie at the heart of PCD analyses and omissions and errors can undermine the research findings by misclassifying patients with or without conditions of interest, hence they should be treated as an integral part of the methods in such analyses and always disclosed to ensure replicability.[11]

Although some guides exist for the creation of such code-lists,[12] there is generally little information available, especially for specific conditions. In addition, there is not necessarily one definitive approach, and different approaches might be better suited to different questions and scenarios. The methods associated with primary care database analyses are also very complex, and researchers often have to compromise due to word restrictions when they are reporting their work in clinical journals. Consequently, the details on code-list creation (and usually the code-lists themselves) are often not included, despite being vital elements of study methodology.

We attempt to address this gap by presenting the methodology we have developed to answer numerous clinical and policy questions in detailed steps,[13–20] while we also provide *pcdsearch* a finalised command in both Stata and R that is an integral part of the process. We use severe mental illness (SMI) as an exemplar, but the method is not condition-specific and should be relevant to any condition. The end product of the method was subsequently used to extract a dataset of SMI cases from the Clinical Practice Research Datalink (or CPRD; formerly known as the General Practice Research Database or GPRD), one of the largest validated PCDs in the world, and used to provide insight into the comorbidity burden and consultation rates of this patient group.[8, 10]

## Methods

### Read codes

Read codes are alphanumeric labels that represent unique clinical concepts, developed by GP Dr James Read in the 1980s.[21] The ICD-9 classification in use at the time in UK hospitals was used as a starting point in the development of a coding system specific to primary care.[22] By 1986 a single system had been developed in collaboration with Abies Informatics Ltd.[23] The new system offered standardisation and was broad, comprehensive and hierarchical, yet easy to use. Over 98,000 Read codes exist, some of which highlight Dr Read’s sense of humour (e.g. 13HV400: Seven year itch—marital), and became the backbone of the UK general practice computerisation which was complete by the early 2000s.[5] All UK general practices use the coding system, and it currently forms the basis of the QOF business rules.[24, 25]However, plans exist for its withdrawal by 2020 and replacement by SNOMED CT, a standard clinical terminology for the NHS to be used across all care settings and clinical domains.[26]

### Process

**Fig 1:**
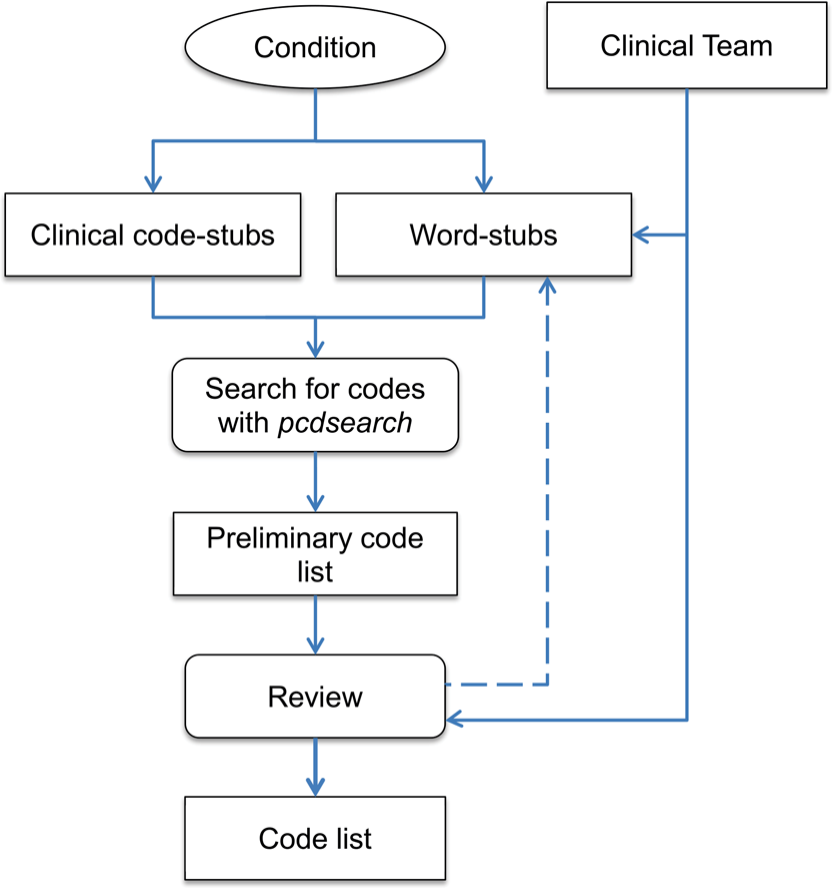
Process flowchart The first step is the definition and delineation of the condition. Within UK primary care, it is also important to consider whether the condition is one of those incentivised under the QOF, since a specific set of business rules will be available for use as a starting point. An expert panel, consisting of clinicians with experience in the particular condition and primary care clinical systems, should suggest a set of search key-words, key-phrases and codes (QOF-specific or not). In the context of a primary care database like the CPRD, the search is focused on two lookup files which contain codes and descriptions for clinical events (mainly diagnoses and referrals) and products (mainly drugs). To facilitate the search we have created *pcdsearch* a Stata/R command that can automate this aspect of the process, implementing advanced search rules which we describe in the next section.

In this section we describe the process we designed to generate a code list associated with a particular medical condition. Although it can be applied to any condition and coding system, provided the condition is available within the respective system, we focus on the generation of a Read code list to identify patients with SMI in UK general practice. The methodology is summarised in a flowchart in Fig 1.

The code lists produced by *pcdsearch*, one for clinical events and one for medicinal products, will include many false positives. For example, searching for “stroke” will also return “sunstroke”, and this inclusive list is reviewed by the expert panel to select a subset through consensus. Occasionally, additional key-words or even codes might be identified at this stage and the process repeated. After any such iterations, the final product is a code list that can be used in dedicated statistical software like Stata or R to identify patients with the condition of interest in the primary care database.

The final code lists need to be conservative, with each code on the selected list associated with the condition of interest and with that alone. Researchers can afford to be conservative at this stage, since patients will very likely be associated with numerous codes that denote a particular condition, especially if chronic, and only one such code can flag a patient (although researchers can apply stricter criteria, for example the presence or Read codes and drugs to accept the presence of a condition). If a patient is not linked with any conservative codes but only with an ambiguous code which indicates that the patient might or might not have the associated condition, then he or she is likely to be condition-free. QOF codes should be treated as conservative, except in the case of exception reporting codes which designate a patient as being unsuitable for the quality indicators.[27] Therefore, a conservative code-list should be a superset of the relevant QOF code-list.

Sometimes, however, full agreement amongst clinicians on the final list is not achieved or a set of additional speculative codes may need to be investigated in addition to the agreed conservative list. For example, some codes, although ambiguous and not exclusive to the condition of interest, might strongly indicate its presence. In such a scenario, a sensitivity list might be produced including both “conservative” and “speculative” codes or fully agreed and partly agreed codes, to reflect the uncertainty in the selection process. This code-list will be a superset of the conservative code-list.

### Search algorithm

We created *pcdsearch* a Stata/R command to automate the search process. It encompasses a high sensitivity and low specificity approach, with the aim of not missing any relevant codes. The command produces two intermediate code lists (clinical and product), which the expert panel can review and finalise. The Stata command is available for download through the SSC archive or the senior author’s personal website, by typing ‘ssc install pcdsearch’ or ‘net from http://statanalysis.co.uk’ within the Stata environment. The R package is available from the rOpenHealth project on Github (https://github.com/rOpenHealth/rpcdsearch). Details on the functionality of the command are provided with the package help files, and here we will only highlight a few aspects of its flexible search rules.

Clinical codes are searched for exactly as inputted, at the start of the respective field. Hence, search for "H33" would return H331.11 (Late onset asthma) but not 8H33.00 (Day hospital care). More search options are available for word-stubs and *pcdsearch* allows users to search the description fields of clinical events or products for:

1. Single word-stubs which will return all cases that include any form of them. For example, "angin" will return "Ludwig's angina" (Read code J083300) but also "Head-banging" (Read code E273100).
2. Exact phrases, using underscores to separate word-stubs. For example, "ischemic_cardiomyopathy" will search for "ischemic cardiomyopathy", with the same rules as for single word-stubs.
3. Two or more word-stubs anywhere within the description field, separating them with plus signs. For example, "alcohol+depend" will return cases where both "alcohol" and "depend" are encountered within a single description field, with the same rules as for single word-stubs.
4. Single word-stubs, preceded by a dollar sign define exclusions. For example, using "splen" and "$hypersplenism" will return cases including "splen" but not any with "hypersplenism". This option is there to help users reduce the number of false-positives and produce code lists that are easier to review.

### Application to SMI

Next we apply the methodology described above to extract Read codes associated with severe mental illness (SMI), from the CPRD database, which contains complete anonymised medical records from UK primary care. SMI is not straightforward to delineate and different definitions exist, but it generally refers to illnesses associated with psychosis. This psychosis-based approach is used by the QOF business rules to generate SMI registers, which include patients with schizophrenia, bipolar disorder, affective disorder and other types of psychosis.[28] The study was approved by the independent scientific advisory committee (ISAC) for CPRD research (reference number: 12_123R). No further ethics approval was required for the analysis of the data.

## Results

### Code-list generation

**Fig 2:**
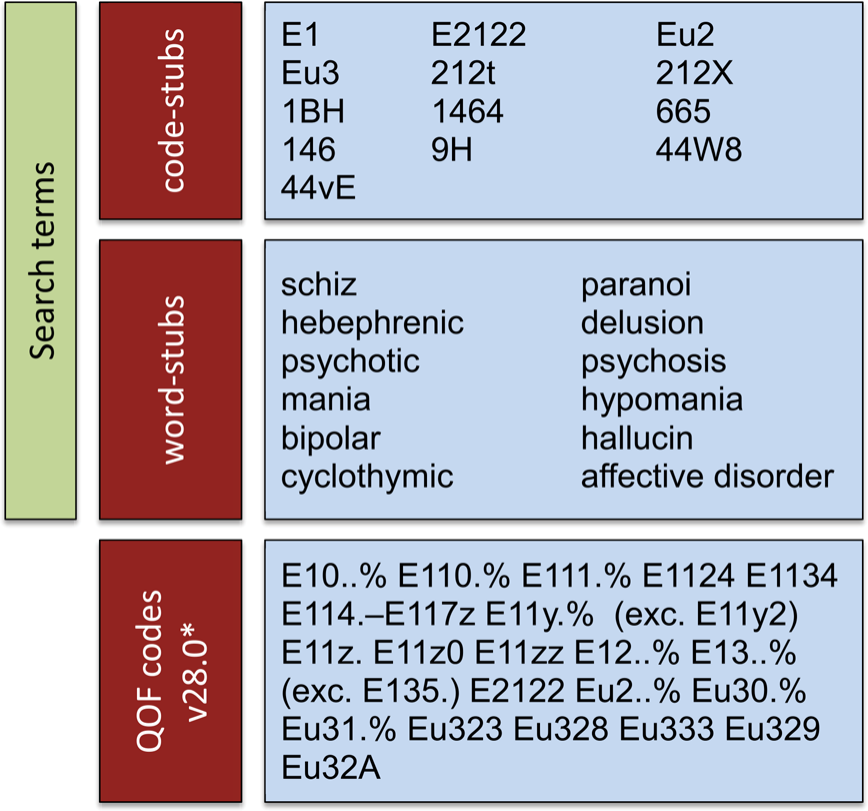
Search terms used with *pcdsearch* to obtain the intermediate SMI code-list*. * QOF codes are not directly used in the search algorithm but are used to inform the code-stubs to be used.

Although certain classes of drugs are associated with SMI, largely antipsychotics and antidepressants, they are not prescribed exclusively to patients with the condition. For example, antipsychotics have also been used to manage behavioural disturbances in patients with dementia, and antidepressants have a number of licensed indications, including neuropathic pain relief. An antipsychotic or antidepressant prescription therefore cannot identify an SMI patient with certainty, and for this reason we did not use product prescriptions in the process. This is not always the case, however, and products might be associated exclusively with a particular condition (for example, insulin with diabetes). Therefore, our search strategy focused on Read codes and their descriptions (Fig 2). Using the QOF business rules for SMI as a starting point, we identified relevant code-stubs to be used in the process. In addition, the clinicians in our team generated a list of word-stubs to be searched for in the description field of Read codes. Any codes thus identified could potentially be linked to SMI diagnosis or care.

**Fig 3:**
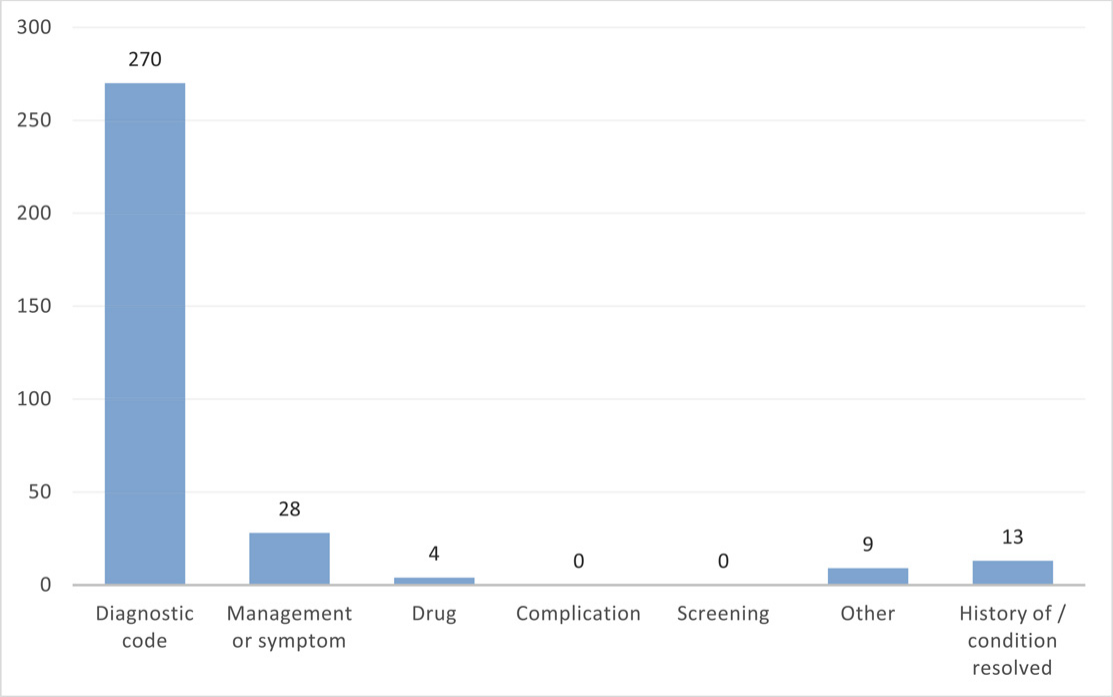
Frequencies for code categories in the final Read code-list.

Using the *pcdsearch* command in the June 2012 version of CPRD Gold with the word- and code-stubs specified in Fig 2 returned 506 potentially relevant Read codes (SMI code-lists). This extended code-list was independently reviewed by the clinical experts in our team and consensus was reached on relevant diagnostic codes, used to directly define SMI, as well as other categories of interest (management or symptom, drug, complication, screening, history of the condition or resolved, and other). A total of 270 diagnosis codes for SMI were agreed on after review, which comprised our “conservative” code-list with which to identify cases that almost definitely have the condition (except for any misdiagnoses and recording mistakes). A more “speculative” code-list could also be generated, including additional code categories besides diagnostic. Frequencies for the code identified as relevant or potentially relevant, by category, are provided in Fig 3. The generated code-lists are available online on the www.clinicalcodes.org repository.[11]

### SMI cases extraction

**Fig 4:**
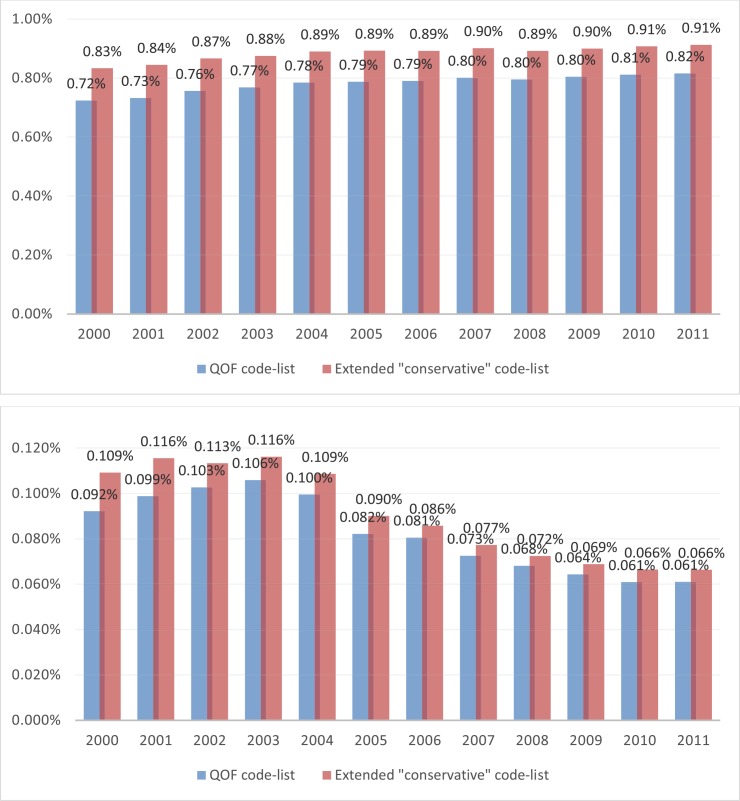
SMI Prevalence (top) and incidence (bottom) rates over time, using QOF and conservative code lists.

**Fig 5:**
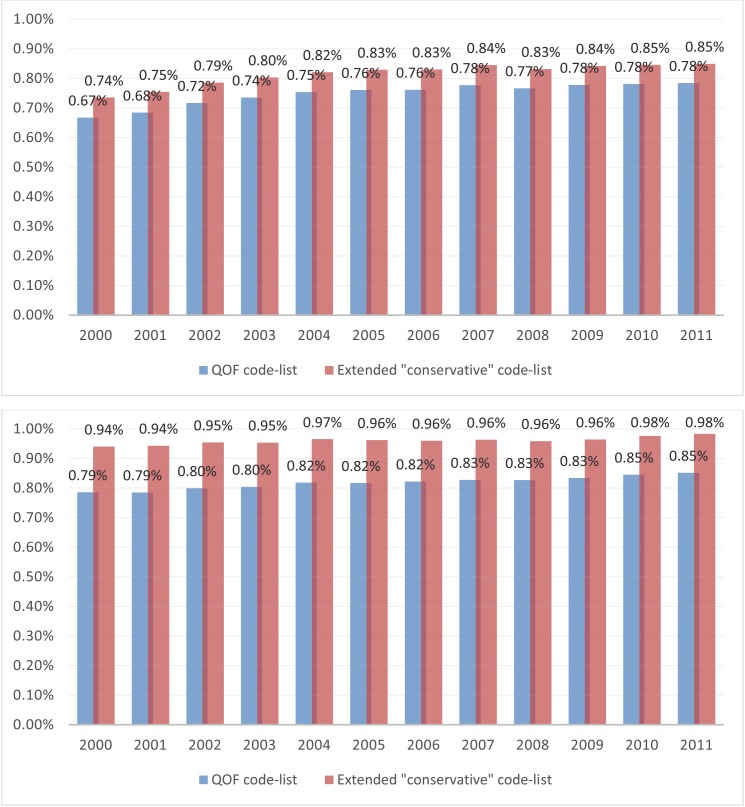
SMI Prevalence rates over time using QOF and conservative code lists, for men (top) and women (bottom).

Both the QOF and conservative code-lists were then applied to clinical and referral files in the June 2012 version of the CPRD Gold database, to obtain cases associated with an SMI diagnosis. Details about the number of cases extracted by financial year are provided elsewhere.[8, 10] Here we focus on the comparison between the two approaches, in terms of the differences observed in SMI prevalence and incidence rates over time (Fig 4). The rates obtained with the conservative code-list are considerably higher than those obtained with the QOF code-list alone, indicating that the latter approach might be missing a not negligible number of SMI cases, especially for earlier years. The greatest differences were observed in from 2000 to 2004 for prevalence (0.11%) and from 2000 to 2001 for incidence (0.02%). Interestingly, the differences appeared greater in women than in men (Fig 5).

## Discussion

We presented a methodology with which researchers of electronic health records databases in general, and primary care databases in particular, can complete the first step in almost any observational analysis with routinely collected data: the generation of a reliable set of clinical codes with which to identify cases with the diagnosis, or diagnoses, of interest. There are many steps to execute before a final code-list is obtained, and clinical input is required at both the first and last steps of the process. We also provided *pcsearch*, a dedicated Stata/R command that can automate an important part of the process: the inclusive search to identify potentially relevant clinical codes using inputted word- and code-stubs.

In the application of the methodology to SMI, which we used as an example, we demonstrated that over-reliance on sets of codes provided by a policy framework (the UK Quality and Outcomes Framework in this instance) may be problematic, with a considerable number of cases missed leading to under-reporting of the condition of interest. In addition, we observed a larger disparity in the cases obtained under the two approaches (QOF codes only versus a “conservative” code-list from our methodology) for women. Although female SMI incidence and prevalence rates are higher, this effect cannot be explained by that fact alone and a gender effect seems likely.

### Limitations

The suggested methodology is straightforward and easy to use, and supported by the *pcdsearch* command. Some limitations exist, however, which largely pertain to the use of electronic health records databases in general. Clinical code usage evolves over time so code-lists need to be reviewed regularly, especially following major policy interventions with frameworks that utilise clinical codes (such as the Quality and Outcomes Framework). The inclusive nature of the approach ensures that codes are not missed but a large amount of work might be generated, with hundreds or even thousands of codes to be reviewed before a final code-list is agreed upon. As with all observational research with electronic health records, there might be variation between care and recorded care and all clinical information might not be included in the electronic health records. Therefore, complete computerisation in a prerequisite for the application of the generated code list to obtain accurate prevalence or incidence estimates. Misdiagnoses and recording errors are always a potential problem with electronic health record research, but more conservative approaches can provide some protection against these issues, for example, two or more relevant clinical codes might be required to flag a patient as case. Finally, although not a limitation per se, it should be noted that the existence of codes that can describe a condition does not necessarily mean that these codes are used in practice. In our experience with Read codes, a small number of codes are often responsible for most of the identified cases

### Conclusions

We provided a framework and an accompanying command in both Stata and R for researchers of electronic health records databases, with which to identify patients with a particular condition. We used severe mental illness as an example, and identified relevant Read codes to be used with the UK’s CPRD primary care database to estimate prevalence and incidence of the condition. However, the method is invariant to code system or database and can be used with SNOMED CT, ICD or other medical classification code-lists.

## Supporting Information

S1 SMI Code-Lists Code-lists used to define Severe Mental Illness. (XLSX)
